# Pilot Study of Acupuncture Point Laterality: Evidence from Heart Rate Variability

**DOI:** 10.1155/2013/476064

**Published:** 2013-11-27

**Authors:** Guangjun Wang, Yuying Tian, Shuyong Jia, Wenting Zhou, Weibo Zhang

**Affiliations:** Institute of Acupuncture and Moxibustion, China Academy of Chinese Medical Sciences, 16 Dongzhimennei, Nanxiaojie, Dongchen District, Beijing 100700, China

## Abstract

The specificity of acupuncture points (acupoints) is one of the key concepts in traditional acupuncture theory, but the question of whether there is adequate scientific evidence to prove or disprove specificity has been vigorously debated in recent years. Laterality, or the tendency for acupoints on the right or left side of the body to produce different physiological effects, is an important aspect of acupoint specificity. Data is particularly scarce regarding the laterality of the same channel, same-named acupoint located on opposite sides of the body. The aim of this study was to investigate whether Neiguan (PC6) has laterality. A total of eighteen healthy female volunteers were recruited for this study. Electrocardiograms were recorded and heart rate variability was analyzed before, during, and after PC6 was stimulated on either the left or the right side. The results show that during acupuncture, there were significant differences in the standard deviation of RR intervals (STDRR), root mean square of successive differences between RR intervals (RMSSD), and total power between the left PC6 stimulation group and the right PC6 stimulation group, which indicates that PC6 may have laterality.

## 1. Introduction

Acupuncture has been widely used as a healing modality for at least 2500 years [1]. Over the past 30 years, researchers have demonstrated the neurobiological basis for the analgesic effects of acupuncture, which has led to greater acceptance of acupuncture in the scientific community [2, 3]. However, a number of well-designed clinical trials have reported that although “true” acupuncture is superior to usual care, it does not significantly outperform sham acupuncture [1, 4]. These findings are apparently at odds with traditional theories regarding acupuncture point specificity because in traditional Chinese medicine (TCM) theory, acupoint specificity is an essential principle for therapeutic efficacy. On the other hand, some clinical trials [5, 6] and studies using fMRI technology [7, 8] have demonstrated acupoint specificity.

For example, in a previous study performed by our group, we found that ipsilateral stimulation of Hegu (LI4) corresponded to increased blood perfusion in the contralateral Hegu (LI4) [9]. Moreover, the increased degree of blood perfusion was asymmetrical [10], which suggests the laterality, or specificity, of this acupuncture point [11]. In clinical practice, Neiguan (PC6) is one of the most commonly used acupoints and is indicated for treating cardiovascular related disorders in classical texts [12–14]. However, the differences between the bilateral PC6 acupoints have never been scientifically investigated. The aim of this study is to investigate the specificity of bilateral PC6 points based on heart rate variability.

## 2. Materials and Methods

### 2.1. Ethics Statement

This study was reviewed and approved by the Institutional Review Board at the Institute of Acupuncture and Moxibustion, China Academy of Chinese Medical Sciences. Each participant read and signed an informed consent form.

### 2.2. Subjects

18 healthy female volunteers were recruited in this study. All subjects were students from the China Academy of Chinese Medical Sciences and Beijing University of TCM. None of the subjects had a history of prior disease nor had they taken any medication in the six months prior to the study. Each subject was provided with informed consent and had an adequate understanding of the procedure and purpose of this study. Basic characteristics of the participants are shown in Table 1.

### 2.3. Electrocardiogram Measurement Protocol

**Figure 1:**
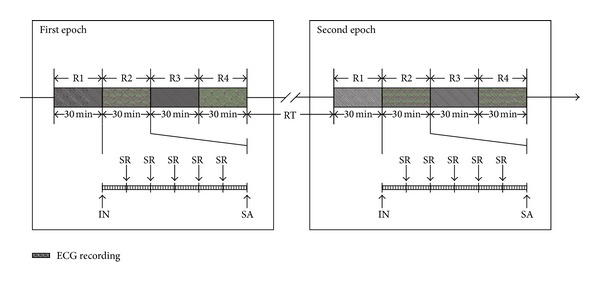
Procedure of acupuncture and measurement. *R*_*i*_  (*i* = 1,2, 3,4): ECG recording time point; IN: insert needle; SA: stop acupuncture; SR: slowly rotate the needle every 5 min (the needle was slowly rotated every 5 min for a total of 30 min during the R2 acupuncture session); RT: rest time between two epochs, the range is 8–22 days.

Before the laboratory procedure began, subjects were placed in a temperature-controlled room (24–26°C) to rest for 10 minutes. The ECG recordings were processed with standard II electrocardiographic lead on NeurOne system (NeurOne, MEGA Electronics Ltd, Finland). The data were digitized with a sampling rate of 500 Hz. In every epoch, the 4 segments of successive ECG were recorded and symbolized as R1 to R4. In each segment, a 30 min ECG recording was obtained using the NeurOne system (shown in Figure 1).

### 2.4. Acupuncture Protocol

For every participant, either the right or left PC6 was stimulated during the first epoch of the study and the opposite side PC6 was stimulated during the second epoch. The stimulus order was determined randomly and the interval time between the two epochs was at least 8 days. For the acupuncture procedure, a small acupuncture needle, 0.25 × 25 mm (100112, Zhen Huan), was gently inserted to a depth of 15 mm in Neiguan (PC6). The needle was slowly rotated every 5 min for a total of 30 min during the acupuncture session in order to maintain the soreness and numbness sensation of De-Qic [9, 15]. The acupuncture process is illustrated in Figure 1.

### 2.5. Data Analysis

The raw data recorded by NeurOne system was exported with ASC format and then imported into Kubio HRV software and analyzed [16]. The analysis parameter was default. In the time domain, the mean heart rate (HR), the standard deviation of RR intervals (STDRR), and the root mean square of successive differences (RMSSD) were analyzed. In the frequency domain, the power spectrum density was analyzed with AR spectrum method in normalized units. The low frequency (LF) and high frequency (HF) were defined as 0.04–0.15 Hz and 0.15–0.4 Hz, respectively. Data are expressed as Mean ± SD. For every recording point, the paired *t*-test was performed between RS group and LS group. The level of significance was defined as *P* < 0.05. Statistical analyses were performed using SPSS (SPSS Inc., Chicago, IL, USA).

## 3. Results

**Figure 2:**
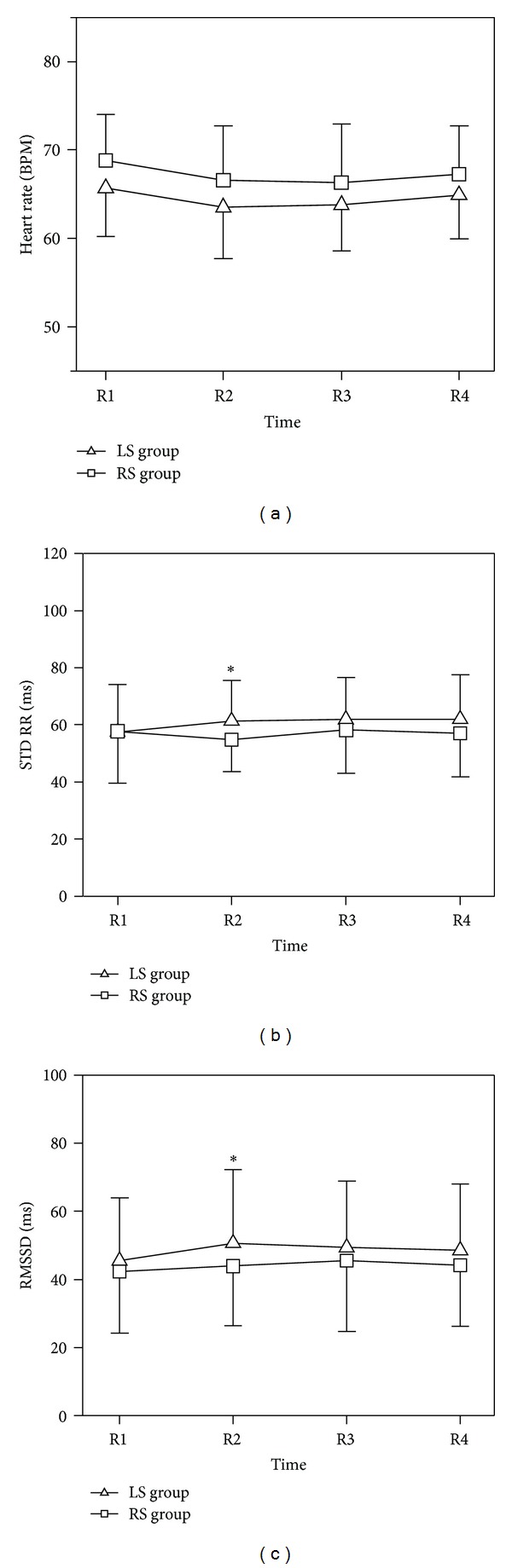
Time domain results. LS: left Neiguan acupoint was stimulated; RS: right Neiguan acupoint was stimulated; STDRR: standard deviation of RR intervals; RMSSD: root mean square of successive differences between RR intervals. Data are expressed as Mean ± SD; *P < 0.05; LS group versus RS group; one-sample paired *t*-test.

Table 1 and Figure 2(a) present the mean value of heart rate during the two epochs of the experiment. HR did not change significantly between the right side (RS) group and left side (LS) group.

### 3.1. Time Domain Results

Table 2 and Figures 2(b) and 2(c) present the results of time domain analysis during the preacupuncture, acupuncture, and postacupuncture periods. The results show that before and after acupuncture (R1, R3, and R4), there were no significant differences in STDRR and RMSSD between the LR-group and RS group. However, during the acupuncture, there were significant differences between the two groups.

### 3.2. Frequency Domain Results

**Figure 3:**
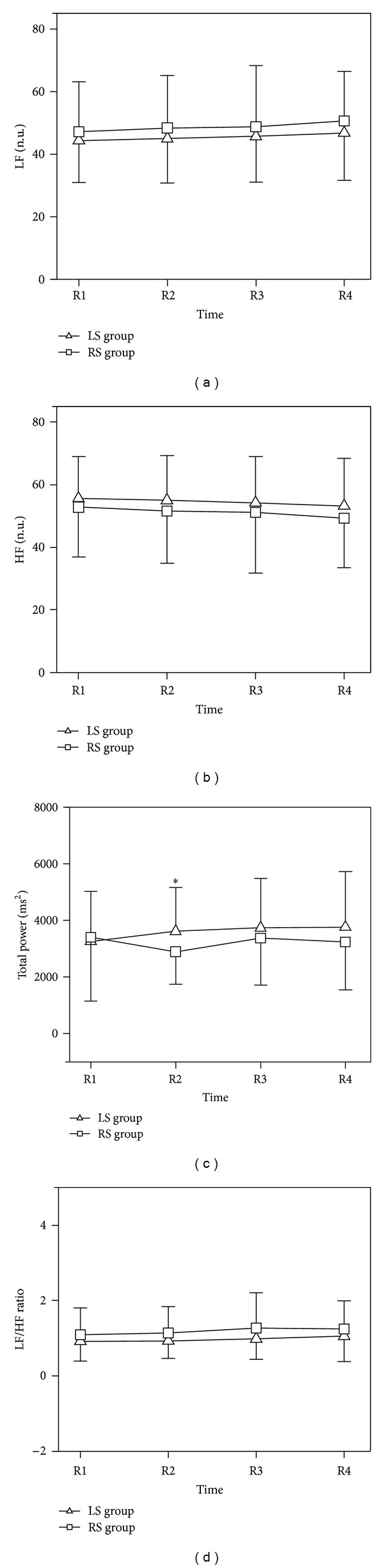
Frequency domain results. LS: left Neiguan acupoint was stimulated; RS: right Neiguan acupoint was stimulated; LF: low frequency; HF: high frequency; n.u.: normalized unit; LF/HF: low frequency high frequency ratio; PSD: Power spectrum density. Data are expressed as Mean ± SD; *P < 0.05; LS group versus RS group; one-sample paired *t*-test.

Table 3 and Figure 3 present the results of frequency domain analysis during the preacupuncture, acupuncture, and postacupuncture periods. The results show that before, during, and after acupuncture (R1, R2, R3, and R4), there was no significant difference in LF, HF, or LF/HF ratio between the LR-group and RS group. However, during the acupuncture, there were significant differences in total power between the two groups.

## 4. Discussion

When a needle is inserted into a point on the body, various neural and neuroactive components are activated [17, 18]. Acupuncture has been shown to have clear central nervous system and autonomic nervous system effects both in humans [19, 20] and in animals [21]. Previous studies showed that manual stimulation of Hegu (LI4) resulted in specific changes in alpha EEG frequency and in HRV parameters. The relationship between the HRV parameters and the special EEG band might point to a specific modulation of cerebral function by acupuncture [22]. On the other hand, power spectral analysis of heart rate variability (HRV) has recently been used as a sensitive index of autonomic nervous system activity. The analysis of HRV provides quantitative information regarding autonomic control mechanisms in the body [23]. For these reasons, HRV has recently been adopted as an index used to evaluate the effects of acupuncture [24].

A previous study indicated that the cardiac modulatory balance differs between genders and is characterized by a greater influence of the autonomic vagal component in women and by the sympathetic component in men [25]. Another study investigated the influence of age and gender on the short-term HRV indices and revealed significant modifications of the indices especially by age but partly also by gender, especially in the younger groups [26]. To exclude gender bias, we only recruited the healthy adult females in this study.

According to the traditional acupuncture theory, acupoints are distributed along meridians and will often have different effects in treatment. PC6 is a classic acupuncture point, and it is considered to be effective in treating cardiovascular disorders. Evidence has recently shown that violet laser stimulation at the PC6 induces a significant increase of total heart rate variability [27].

Previous studies demonstrated that acupuncture manipulation significantly decreased the LF spectral component of HRV and significantly reduced LF/HF, which is an index of sympathetic activity [28]. In the present study, acupuncture effects on heart rate variability mainly occurred in the acupuncture period. After the acupuncture was discontinued, this effect disappeared. Since the effects of acupuncture are the result of central nervous system regulation, we can expect laterality due to the fact that information is processed in the brain, which has hemispheric dominance [29]. In this study, differences in LF, HF, and LF/HF between the two groups were not observed; however, the difference in total power was significant.

Obviously, this is a plot study in acupoint laterality, the conclusions just resulted from the female healthy subjects aged from 21 to 33, and just the PC6 was investigated in the study. So, we cannot be sure whether all acupoints have the laterality, and we also cannot be sure whether this laterality will be changed under different conditions such as disorder, handedness, or aging. In particular, we note that the laterality of PC6 was just based on the HRV analysis. The mechanism and factors analysis needed further research.

## Figures and Tables

**Table 1:** Basic characteristics of the study participants (*n* = 18).

Characters	Mean ± SD	Range
Age (years)	25.61 ± 2.38	33–21
High (cm)	161.78 ± 4.60	172–150
Body weight (kg)	50.50 ± 4.89	60–42
Body mass index (BMI)	19.28 ± 1.58	22.04–15.79
Interval of two measures (days)	13.28 ± 3.99	22–8

**Table 2:** Time domain result of heart rate variability.

	Time	LS group	RS group	*T* value	*P* value
Mean HR (BPM)	R1	65.669 ± 5.439	68.801 ± 5.237	−1.914	0.073
	R2	63.505 ± 5.771	66.561 ± 6.131	−1.886	0.077
	R3	63.752 ± 5.145	66.314 ± 6.581	−1.581	0.132
	R4	64.897 ± 4.970	67.240 ± 5.457	−1.641	0.119

STDRR (ms)	R1	57.495 ± 16.635	57.737 ± 18.111	−0.046	0.964
	R2	61.386 ± 14.168	54.763 ± 11.235	2.238	0.039
	R3	61.886 ± 14.773	58.182 ± 15.163	0.891	0.386
	R4	62.010 ± 15.453	57.088 ± 15.399	1.748	0.099

RMSSD (ms)	R1	45.418 ± 18.573	42.256 ± 18.084	0.993	0.335
	R2	50.629 ± 21.593	43.886 ± 17.490	2.492	0.023
	R3	49.330 ± 19.469	45.540 ± 20.906	1.122	0.277
	R4	48.488 ± 19.496	44.151 ± 17.963	1.714	0.105

LS: left Neiguan acupoint was stimulated; RS: right Neiguan acupoint was stimulated; HR: heart rate; STDRR: standard deviation of RR intervals; RMSSD: root mean square of successive differences between RR intervals; BPM: beat per minute. Data are expressed as Mean ± SD.

**Table 3:** Frequency domain result of heart rate variability.

	Time	LS group	RS group	*T* value	*P* value
LF	R1	44.308 ± 13.443	47.128 ± 16.006	−1.325	0.203
	R2	44.94 ± 14.216	48.373 ± 16.697	−1.429	0.171
	R3	45.693 ± 14.727	48.755 ± 19.521	−0.927	0.367
	R4	46.747 ± 15.164	50.605 ± 15.892	−1.605	0.127

HF	R1	55.618 ± 13.433	52.806 ± 15.981	1.322	0.204
	R2	54.988 ± 14.217	51.548 ± 16.68	1.432	0.170
	R3	54.245 ± 14.728	51.182 ± 19.511	0.927	0.367
	R4	53.184 ± 15.169	49.332 ± 15.889	1.604	0.127

Total Power	R1	3258.025 ± 1758.083	3397.377 ± 2253.850	−0.218	0.830
	R2	3613.889 ± 1537.027	2892.476 ± 1143.268	2.375	0.030
	R3	3738.289 ± 1736.069	3374.885 ± 1663.170	0.781	0.445
	R4	3761.157 ± 1960.836	3241.175 ± 1700.276	1.408	0.177

LF/HF	R1	0.912 ± 0.517	1.087 ± 0.713	−1.634	1.121
	R2	0.928 ± 0.463	1.142 ± 0.693	−2.055	0.056
	R3	0.979 ± 0.547	1.271 ± 0.928	−1.855	0.081
	R4	1.052 ± 0.672	1.247 ± 0.743	−1.627	0.122

LF: low frequency; HF: high frequency; n.u.: normalized unit; LF/HF: low frequency high frequency ratio. Data are expressed as Mean ± SD.
